# The Primary Effect on the Proteome of *ARID1A*-mutated Ovarian Clear Cell Carcinoma is Downregulation of the Mevalonate Pathway at the Post-transcriptional Level*[Fn FN2]

**DOI:** 10.1074/mcp.M116.062539

**Published:** 2016-09-21

**Authors:** Aaron R. Goldman, Benjamin G. Bitler, Zachary Schug, Jose R. Conejo-Garcia, Rugang Zhang, David W. Speicher

**Affiliations:** From the ‡Molecular and Cellular Oncogenesis Program, The Wistar Institute, 3601 Spruce St., Philadelphia, Pennsylvania 19104;; §Gene Expression and Regulation Program, The Wistar Institute, 3601 Spruce St., Philadelphia, Pennsylvania 19104;; ¶Tumor Microenvironment and Metastasis Program, The Wistar Institute, 3601 Spruce St., Philadelphia, Pennsylvania 19104;; ‖The Center for Systems and Computational Biology, The Wistar Institute, 3601 Spruce St., Philadelphia, Pennsylvania 19104

## Abstract

Inactivating mutations in *ARID1A*, which encodes a subunit of the SWI/SNF chromatin-remodeling complex, are found in over half of ovarian clear cell carcinoma cases and more broadly across most types of cancers. To identify ARID1A-dependent changes in intracellular signaling pathways, we performed proteome analyses of isogenic ovarian clear cell carcinoma cell lines with or without ARID1A expression. Knockout of ARID1A in an ovarian clear cell carcinoma cell line with wild-type *ARID1A*, OVCA429, primarily resulted in downregulation of the mevalonate pathway, an important metabolic pathway involved in isoprenoid synthesis, cholesterol synthesis, and other downstream pathways. In a complementary experiment, expression of wild-type ARID1A in an ovarian clear cell carcinoma cell line containing mutated *ARID1A,* OVISE, affected the mevalonate pathway in a reciprocal manner. A striking aspect of these analyses was that, although only 5% of the detected proteome showed significant abundance changes, most proteins in the mevalonate pathway were coordinately affected by ARID1A status. There were generally corresponding changes when comparing the proteomics data to our previously published microarray data for ectopic expression of ARID1A in the OVISE cell line. However, ARID1A-dependent changes were not detected for genes within the mevalonate pathway. This discrepancy suggests that the mevalonate pathway is not regulated directly by ARID1A-mediated transcription and may be regulated post-transcriptionally. We conclude that ARID1A status indirectly influences the mevalonate pathway and probably influences other processes including glycogen metabolism and 14-3-3-mediated signaling. Further, our findings demonstrate that changes in mRNA levels are sometimes poor indicators of signaling pathways affected by gene manipulations in cancer cells.

Epithelial ovarian cancer is the fifth highest cause of cancer mortality among women in the United States with an estimated death toll of 14,240 in 2016 (1). Ovarian clear cell carcinomas (OCCC)^1^ accounts for an average of 5% of all epithelial ovarian cancers with occurrence rates greater than 20% in certain Asian populations (2). OCCC tumors are refractory to standard treatment regimens and carry a poor prognosis at late-stage detection (3).

OCCC cells have a distinct phenotype compared with other ovarian cancer histological subtypes that includes cytosolic glycogen stores that give them their characteristic “clear” appearance and a unique gene signature (4, 5). Loss-of-function mutations in *ARID1A* are found in the majority of OCCCs and one-third of ovarian endometrioid carcinomas (6, 7). *ARID1A* mutations are also prevalent in cancers of other tissues, including subtypes of breast and gastric cancers (8–10). *ARID1A* encodes the ARID1A/BAF250a subunit of the switch/sucrose nonfermentable (SWI/SNF) chromatin remodeling complex, an epigenetic regulator that modulates gene expression and DNA repair through nucleosome repositioning (11, 12). ARID1A may confer sequence specificity to the SWI/SNF complex through its inherent DNA-binding activity (13–16) or via interactions with transcriptional regulators such as p53 (17). Mutation of *ARID1A* likely drives cancer progression by increasing dependence on alternative SWI/SNF complexes (18) with partially overlapping transcriptional profiles (19).

Efforts to uncover the global effects of *ARID1A* mutation in OCCC have thus focused primarily on changes in gene transcription. Mutation of *ARID1A* in OCCC cells results in loss of homeostasis between SWI/SNF and polycomb repressive complex 2, which were found to antagonistically regulate gene expression (20). Restoring wild-type ARID1A or inhibiting EZH2, the catalytic subunit of polycomb repressive complex 2, inhibits Phosphoinositide 3-kinase/AKT signaling through up-regulation of *PIK3IP1*. Profiling of ARID1A-deficient tumors in a mouse ovarian cancer model showed enrichment for genes associated with mesenchymal-epithelial transition (21). However, steady-state protein levels are often better indicators of the functional state of the cell (22). A preliminary targeted proteome approach using reverse phase protein arrays profiled 31 OCCC tumor samples with known *ARID1A* mutational status using 116 antibodies and observed differential expression of pAKT-Thr^308^ (23).

To better understand the global impact of *ARID1A* loss-of-function mutations on intracellular signaling networks, we assessed changes in the proteome because of ARID1A knockout in a *ARID1A* wild-type OCCC cell line, OVCA429, in an unbiased, in-depth manner using high resolution LC-MS/MS. Although *ARID1A* status had a minimal impact on the proteome overall, extensive effects on specific metabolic signaling pathways were observed. Notably, enzymes that function in the mevalonate pathway, which is involved in critical processes such as cholesterol biosynthesis and protein prenylation, showed decreased protein levels when ARID1A expression was abrogated. We validated these findings in an *ARID1A*-mutated OCCC cell line, OVISE, and observed an increase in abundance of mevalonate pathway enzymes when wild-type ARID1A was ectopically expressed. These results suggest a potential mechanism by which *ARID1A* mutation may contribute to OCCC progression and provides potential therapeutic targets for this difficult to treat form of ovarian cancer.

## EXPERIMENTAL PROCEDURES

### 

#### 

##### Cell Lines and 2D Culturing Conditions

OVCA429 parental cell line and OVISE cell line expressing tetracycline-inducible wild-type ARID1A (17) originated from the laboratory of I. M. Shih. Cell lines were tested for mycoplasma (University of Pennsylvania School of Medicine, Cell Culture Services). All cell lines were cultured on polystyrene in a 2D format in the presence of 5% CO_2_ at 37 °C. OVCA429 cells were maintained in RPMI 1640 (Corning, Corning, NY, cat. no. 10–092-CM) supplemented with 10% heat-inactivated FCS (Sigma-Aldrich, St. Louis, MO, cat. no. F4135). OVISE cells expressing inducible wild-type ARID1A were maintained in RPMI 1640 supplemented with 10% Tet System Approved FCS (Clontech, Mountain View, CA, cat. no. 631107). All media contained 1% Penicillin-Streptomycin (Corning, cat. no. 30-002-CI).

##### Construction and Use of CRISPR Plasmids

The Clustered regularly interspaced short palindromic repeats (CRISPR) plasmids were a kind gift from Dr. Cigall Kadoch. *ARID1A*-specific guide RNA (gRNA; 5′-CGGGTTGCCCAGGCTGCTGGcgg-3′) was inserted into pSpCas9 (BB)-2A-Puro (PX459) using AgeI/EcoRI. Fugene6 (Promega, Madison, WI) transfection agent was utilized to transfect plasmid into cells and cells were selected with puromycin (1 μg/ml). Isogenic clonal cell lines were established based on ARID1A protein expression.

##### Immunoblotting

Primary antibodies used for immunoblots were ARID1A (Cell Signaling, Beverly, MA, cat. no. 12354) at 1:1000 and GAPDH (Sigma-Aldrich, cat. no. G8795) at 1:100,000. Secondary antibodies were Peroxidase-conjugated Anti-Rabbit IgG (Sigma-Aldrich, cat. no. A0545) at 1:50,000 and Peroxidase-conjugated Anti-Mouse IgG (Sigma-Aldrich, cat. no. A9044) at 1:50,000.

##### Sample Preparation for Proteome Analysis

OVCA429 cells were plated on tissue culture-treated Petri dishes (Corning, cat. no. 430599). OVISE cells harboring inducible wild-type ARID1A were similarly plated and treated with either DMSO or 1 μg/ml doxycycline in DMSO to induce ARID1A expression (96 h treatment duration, refreshed after 72 h). Cells were harvested at 60–80% confluency. Cell lysates were prepared using SDS lysis buffer (50 mm Tris-HCl pH 7.5, 150 mm NaCl, 1% SDS, 1 mm EDTA) with freshly added 1 mm DTT and protease inhibitors (150 μm PMSF, 1 μg/ml Pepstatin A, and 1 μg/ml Leupeptin). Lysates were sonicated briefly to shear genomic DNA. Protein content of clarified lysates was quantified by Pierce BCA Protein Assay Kit (Thermo Fisher Scientific, Boston, MA, cat. no. 23227). For proteome analyses, 12.5 to 25 μg lysate was separated on 10% Bis-Tris NuPAGE minigels (Thermo Fisher Scientific, cat. no. NP0301) with MES running buffer (Thermo Fisher Scientific, cat. no. NP0002) until the dye front migrated 0.5 cm. In-gel trypsin digestion was performed as described previously (24).

##### Proteome Analysis

LC-MS/MS of tryptic peptides was performed using a nanoACQUITY UPLC (Waters, Milford, MA) in-line with a Q Exactive Plus or Q Exactive HF mass spectrometer (Thermo Fisher Scientific). Typically, 0.25–1 μg of each tryptic digest, estimated assuming a 50% recovery of the amount of protein loaded into the gel lane, was loaded onto a 180 μm x 20 mm nanoACQUITY UPLC Symmetry C18 trap column with 5 μm particle size and 100 angstrom pore size (Waters, cat. no. 186006527) with 0.1% formic acid in Milli-Q water (solvent A). Analytical separation was performed on a 1.7 μm x 250 mm nanoACQUITY UPLC Peptide BEH C18 column with 1.7 μm particle size and 130 angstrom pore size (Waters, cat. no. 186003546) using a 245 min gradient with 0.1% formic acid in acetonitrile (solvent B) as follows: 5–30% B over 225 min, 30–80% B over 5 min, and constant 80% B for 15 min. A blank was run between each sample to minimize carryover by injecting water and using a 30 min gradient with the same solvents. Peptides were analyzed using the same parameters for the Q Exactive Plus and HF instruments unless otherwise indicated. Full MS spectra were recorded at a resolution of 70,000 for the Plus and 60,000 for the HF with a scan range of 400–2000 *m*/*z* in profile mode. Full MS automatic gain control target and maximum injection time were set to 3e6 and 50 ms, respectively. MS2 spectra were recorded at 17,500 resolution for the Plus and 15,000 resolution for the HF. MS2 automatic gain control target and maximum injection time were set to 5e4 and 50 ms, respectively. Data-dependent analysis was performed on the 20 most abundant ions using an isolation width of 1.5 *m*/*z* and an underfill of 1%, corresponding to a minimum threshold of 1e4. Peptide match was set to preferred, and unassigned and singly charged ions were rejected. Dynamic exclusion was set to 30 s for the Plus and 45 s for the HF.

##### Experimental Design and Statistical Rationale

All experimental and control samples were analyzed using biological triplicates to allow for robust statistics when performing label-free quantitative comparisons. This is more advantageous than technical replicates as it accounts for variability between cell culture plates and at all stages of sample preparation and processing. The OVCA429 and OVISE proteome comparisons each generated 6 RAW files, 3 corresponding to experimental conditions and three corresponding to control, that contain all acquired Full MS and MS2 spectra. Base peak chromatograms were inspected visually in Xcalibur Qual Browser version 3.0.63 (Thermo Fisher Scientific). RAW files were processed by MaxQuant version 1.5.1.2 using default parameters unless otherwise specified (http://www.maxquant.org) (25). All RAW files for a given parental cell line were analyzed together in a single MaxQuant run. Database searches were performed using the Andromeda search engine included with the MaxQuant release (26) with the UniProt human sequence database (July 28, 2014; 145,433 sequences; 53,453,851 residues) and an in-house contaminants database of common laboratory contaminants, including keratins, bovine proteins detected in FCS, trypsin, and mycoplasma proteins to detect potential mycoplasma contamination of cell cultures (Sequences obtained from UniProt, July 28, 2014; 3671 sequences; 1,338,375 residues). Precursor mass tolerance was set to 4.5 ppm in the main search, and fragment mass tolerance was set to 20 ppm. Digestion enzyme specificity was set to Trypsin/P with a maximum of 2 missed cleavages. A minimum peptide length of 7 residues was required for identification. Up to 5 modifications per peptide were allowed; acetylation (protein N-terminal) and oxidation (Met) were set as variable modifications, and carbamidomethyl (Cys) was set as a fixed modification. No Andromeda score threshold was set for unmodified peptides. A minimum Andromeda score of 40 was required for modified peptides. Peptide and protein false discovery rates (FDR) were both set to 1% based off a target-decoy reverse database. Proteins that shared all identified peptides were combined into a single protein group. If all identified peptides from one protein were a subset of identified peptides from another protein, these proteins were also combined into that group. Peptides that matched multiple protein groups (“razor” peptides) were assigned to the protein group with the most unique peptides. “Match between runs” based on accurate *m*/*z* and retention time was enabled with a 0.7 min match time window and 20 min alignment time window. Label-free quantitation (LFQ) was performed using the MaxLFQ algorithm built into MaxQuant (27). Briefly, peaks were detected in Full MS, and a three-dimensional peak was constructed as a function of peak centroid *m*/*z* (7.5 ppm threshold) and peak area over time. Following de-isotoping, peptide intensities were determined by extracted ion chromatograms based on the peak area at the retention time with the maximum peak height. Peptide intensities were normalized to minimize overall proteome difference based on the assumption that most peptides do not change in intensity between samples. Protein LFQ intensities were calculated from the median of pairwise intensity ratios of peptides identified in two or more samples and adjusted to the cumulative intensity across samples. Quantification was performed using razor and unique peptides, including those modified by acetylation (protein N-terminal) and oxidation (Met). A minimum peptide ratio of 1 was required for protein intensity normalization, and “Fast LFQ” was enabled.

Data processing and cluster analysis was performed using Perseus version 1.5.0.31 (http://www.perseus-framework.org) (28), and statistical analysis was performed using Excel 2013 and 2016 (Microsoft, Redmond, WA). Contaminants and protein groups identified by a single peptide were filtered from the data set. FDR was calculated as the percentage of reverse database matches out of total forward and reverse matches. Protein group LFQ intensities were log_2_ transformed to reduce the effect of outliers. For cluster analysis and statistical comparisons between proteomes, protein groups missing LFQ values were assigned values using imputation. Missing values were assumed to be biased toward low abundance proteins that were below the MS detection limit, referred to as “missing not at random” (29), an assumption that is frequently made in proteomics studies (28). The missing values were replaced with random values taken from a median downshifted Gaussian distribution to simulate low abundance LFQ values (demonstrated in supplemental Fig. S4). Imputation was performed separately for each sample from a distribution with a width of 0.3 and downshift of 1.8. Hierarchical clustering was performed on Z-score normalized, log_2_ LFQ intensities using Euclidean distance and average linkage with k-means preprocessing (300 clusters). Log ratios were calculated as the difference in log_2_ LFQ intensity averages between experimental and control groups. Two-tailed, unpaired, homoscedastic Student's *t* test calculations were used in statistical tests as histograms of LFQ intensities showed that all data sets approximated normal distributions. *p* < 0.05 was considered statistically significant. Base 10-fold-change values for ratios < 1 are represented as negative reciprocals of the ratios.

##### Data Availability

The mass spectrometry proteomics data have been deposited to the ProteomeXchange Consortium (http://proteomecentral.proteomexchange.org) via the MassIVE partner repository (http://massive.ucsd.edu/ProteoSAFe/static/massive.jsp) with the data set identifier PXD004570.

##### Comparison of Proteomics and Microarray Data Sets

Microarray data for OVISE cells with and without *ARID1A* induction were obtained from the Gene Expression Omnibus database, accession number GSE54979 (20). Protein groups from the proteomics analysis were mapped to Illumina microarray probes based on Gene Name and Ensembl Gene Accession Number. When duplicate probes were present for a single gene, the probe with the most significant *p* value was selected.

##### Canonical Pathway Analysis

Pathway analysis was performed using Qiagen's (Valencia, CA) Ingenuity Pathway Analysis (IPA) using default parameters unless otherwise specified (http://www.ingenuity.com/pa/). Tab-delimited files containing matrices of identified proteins and associated log ratios and *t* test *p* values were uploaded into the IPA service as “Ingenuity File Format A or B.” Proteins were mapped from UniProt gene names to “IPA IDs” based on “Gene symbol - human.” Unmapped proteins were reconciled based on alternative gene names if possible. Core Analyses were performed using a log ratio threshold of 1 and *p* value threshold of 0.05. Proteins that increased or decreased in abundance were considered. The entire imported data set for a given comparison was used as a reference set in statistical calculations.

## RESULTS

### 

#### 

##### Generation of Isogenic ARID1A Knockout and ARID1A Wild-type OCCC Cell Lines for Proteome Analysis

To assess the impact of *ARID1A* frameshift or nonsense mutations typically found in OCCC (6, 7), we performed proteome comparisons between cells that express wild-type ARID1A and cells that have lost functional ARID1A expression through mutation. However, distinct *ARID1A* wild-type and *ARID1A*-mutated OCCC cell lines have genetic and epigenetic differences that may obfuscate the contribution of *ARID1A* mutational status to proteome differences (20). To address this concern, we utilized CRISPR gene editing to knockout ARID1A expression in the OCCC cell line OVCA429 (30), which expresses wild-type ARID1A protein at a level representative of the majority of commonly used *ARID1A* wild-type OCCC cells lines (17). ARID1A knockout resulted in the loss of detectable ARID1A by immunoblot (supplemental Fig. S1). There were no significant phenotypic effects on cell morphology or cell proliferation (data not shown).

##### Proteome Analysis of ARID1A Knockout in OVCA429

We performed proteome analysis to elucidate the effects of ARID1A loss in OVCA429 using total cell lysates in biological triplicates from ARID1A knockout and control cells. LC-MS/MS was performed on a Thermo Q Exactive Plus mass spectrometer with an unfractionated, 4-hour run per sample. Data from biological triplicates of knockout and control cells were analyzed together in MaxQuant using a protein and peptide FDR of 1% and “matching between runs” based on accurate mass and retention time. A total of 54,037 tryptic peptides were identified when acetylation (protein N-terminal) and oxidation (Met) were counted separately from unmodified versions of the 50,365 sequence unique peptides that were identified across all runs (supplemental Table S1). These peptides were assigned to 5,681 distinct proteins groups. Single peptide hits were filtered from the data set to yield a list of 4,973 high confidence protein groups identified at 0.52% FDR (supplemental Table S2). These protein groups were the focus of all analyses. An average of 10 peptides were identified per protein group, yielding an average sequence coverage of 29%. The depth of proteome analysis was estimated to be 50% based on the expected approximate 10,000 proteins expressed by a cancer cell line at a given time (31).

##### Label-free Comparisons Between ARID1A Knockout and Control Proteomes

**Fig. 1.**
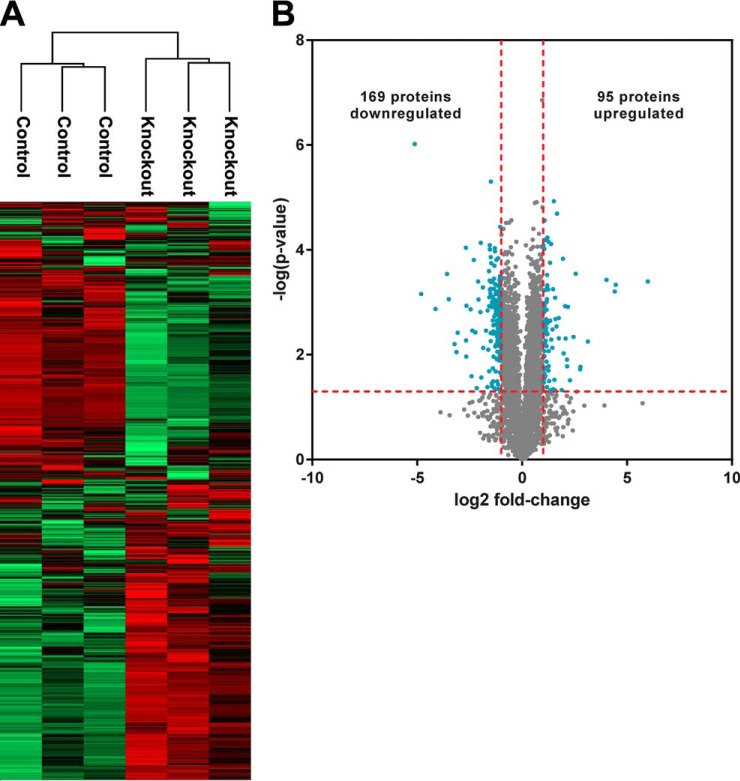
**Characterization of the OVCA429 proteome with or without ARID1A CRISPR knockout.** *A*, Two-way, unsupervised hierarchical clustering of OVCA429 proteomes with and without ARID1A knockout based on LFQ intensities. *B*, Volcano plot comparing ARID1A knockout and control proteomes. Log ratios of LFQ intensities in ARID1A knockout *versus* control were plotted against negative log *p* values from the Student's *t* test based on biological triplicates. *Vertical lines*: fold-changes of ± 2. *Horizontal line*: Student's *t* test *p* of 0.05. *Blue points*: proteins that meet both criteria for significant change between ARID1A knockout and control (i.e. fold-change in abundance > 2 and *p* < 0.05). *Gray points*: proteins that do not meet both of these criteria.

We used label-free quantitation (LFQ) to compare relative abundances of protein groups across the ARID1A knockout and control proteomes. The MaxLFQ algorithm built into the MaxQuant utilizes maximal ratios of MS signal intensities for peptides identified in multiple samples coupled with delayed normalization to allow for accurate comparisons across proteomes (27). The overall effect of loss of ARID1A protein expression was assessed using a global analysis of the biological triplicate knockout and control proteomes. Unsupervised hierarchical clustering based on LFQ intensities resulted in independently clustered knockout and control proteomes, indicating that ARID1A status is the main determinant of differences between the proteomes (Fig. 1*A*). We also compared different passages for each cell line based on average LFQ intensities of protein groups from biological triplicate samples. As has been observed in prior studies, low abundance proteins had a wider spread of signal intensities due to less accurate quantitation near the noise threshold (32). However, there was excellent overall reproducibility between the different culture passage numbers (R^2^ of 0.9803 and 0.9873 for control and knockout cells, respectively), which demonstrates that independent sample preparation had minimal effects on the proteomes (supplemental Fig. S2).

##### ARID1A Knockout Has a Low Overall Impact on the Proteome

We examined the specific proteins affected by ARID1A knockout through quantitative proteome comparisons. Protein groups that significantly changed in abundance as a function of ARID1A status were selected based on magnitude of changes between mean LFQ intensities of the biological replicates as well as the statistical significance of the changes to increase precision because fold-change alone is sensitive to outliers (27). A minimum fold-change of 2 in either direction was required based on the rationalization that smaller changes are less likely to exert a biological effect. A Student's *t* test *p* value of less than 0.05 was used as a statistical cutoff. When comparing the ARID1A knockout and control proteomes, 430 and 2606 proteins satisfied the fold-change and statistical criteria, respectively (Table S3). Combining these relatively relaxed stringency criteria selected only 264 proteins, corresponding to 5% of the identified proteins, as significantly changed in level in the knockout relative to the control (Fig. 1*B*, supplemental Table S3). Of these, 95 proteins increased in abundance and 169 proteins decreased in abundance. ARID1A protein was undetectable in the *ARID1A* wild-type control proteome, likely due to its low abundance. Other components of the BRG1-associated factor (BAF) SWI/SNF complex (33), including SMARCC1/2, SMARCD1/2, DPF2, SMARCE1, SMARCA4, and SMARCB1, were detected but did not exhibit ARID1A-dependent changes. ARID2, a subunit exclusive to the polybromo BRG1-associated factor (PBAF) SWI/SNF complex, significantly increased in level in the knockout, suggesting possible compensation for ARID1A loss via an alternative SWI/SNF complex with an overlapping transcriptional profile (19).

##### ARID1A Knockout Primarily Affects Enzymes Involved in the Mevalonate Pathway

**Fig. 2.**
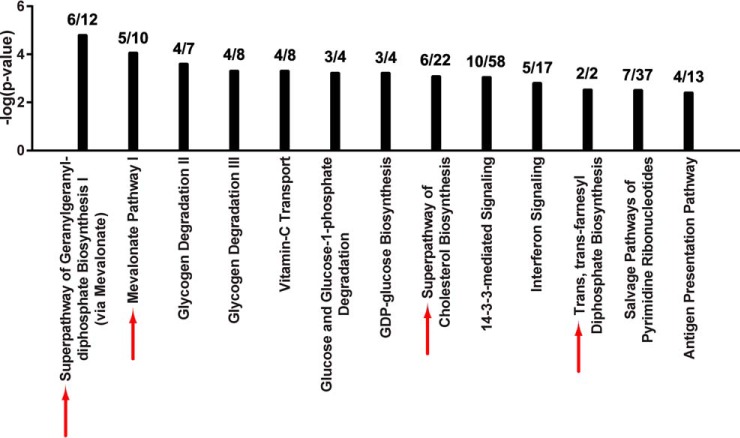
**Knockout of ARID1A downregulates the mevalonate pathway.** Top scoring canonical pathways associated with ARID1A knockout in OVCA429 are shown (IPA, *p* < 0.005 by Fisher's exact test right-tailed). *Black bars*: negative log *p* values for each canonical pathway. The ratio of annotated proteins that significantly changed in level in ARID1A knockout to total identified proteins for a given canonical pathway is shown above each bar. *Red arrows*: canonical pathways that include the mevalonate pathway and are also significantly enriched among proteins that changed upon ARID1A induction. Refer to Fig. 4.

To uncover signaling pathways that are perturbed when ARID1A expression is lost, we performed canonical pathway analysis (IPA) on the set of 264 proteins groups that significantly changed between ARID1A knockout and control, of which 257 were curated in the IPA knowledgebase. Interestingly, a number of metabolic pathways were significantly overrepresented in the ARID1A-dependent proteins compared with all identified proteins (Fig. 2). Four of the 11 highest scoring canonical pathways were related to the mevalonate pathway, which synthesizes a variety of isoprenoids derived from acetyl-CoA that are required for diverse cellular processes, including cholesterol, prenyl groups (geranylgeranyl pyrophosphate and farnesyl pyrophosphate), dolichol, ubiquinone, and heme A (34, 35). Associated pathways included: “Superpathway of Geranylgeranyl-diphosphate Biosynthesis I (via Mevalonate),” “Mevalonate Pathway I,” “Superpathway of Cholesterol Biosynthesis,” and “Trans, trans-farnesyl Diphosphate Biosynthesis.” Coverage of these pathways was extensive with 27.3% to 100% of identified pathway components significantly changing in the knockout proteome (Fig. 2). In total, 6 enzymes in the mevalonate pathway (ACAT2, HMGCS1, MVK, MVD, IDI1, and FDPS) decreased in abundance in the ARID1A knockout (Table I). Proteins relevant to the mevalonate biosynthesis pathway (listed in Table I) were identified in all knockout and control proteomes or not identified in any sample; therefore, imputation of missing values did not affect the calculated fold-changes and *p* values (supplemental Tables S2 and S3). Pathways associated with glycogen and glucose metabolism were also among the top scoring pathways, including “Glycogen Degradation II,” “Glycogen Degradation III,” “Glucose and Glucose-1-phosphate Degradation,” and “GDP-glucose Biosynthesis.” Within these pathways there was a significant decrease in abundance of phosphoglucomutase (PGM1, PGM2, and PGM3) and phosphorylase (PYGL) which should reduce glycogen degradation (Fig. 2). As noted above, the clear cell morphology is because of excessive glycogen accumulation in the cytosol. Another high scoring pathway was “14–3-3-mediated signaling.” 14–3-3 proteins interact with target proteins to regulate a diverse array of signaling pathways (36). Of the 7 human isoforms (β, γ, ϵ, η, σ, τ, and ζ), 14–3-3 σ (SFN) and 14–3-3 τ (YWHAQ) were significantly decreased in the ARID1A knockout relative to control (supplemental Table S3). Loss of 14–3-3 σ in particular frequently occurs in cancer and is associated with metabolic changes that promote cancer progression (36, 37). Similar results were obtained from analysis of a second ARID1A CRISPR knockout clone (supplemental Fig. S1, data not shown).

**Table I:**
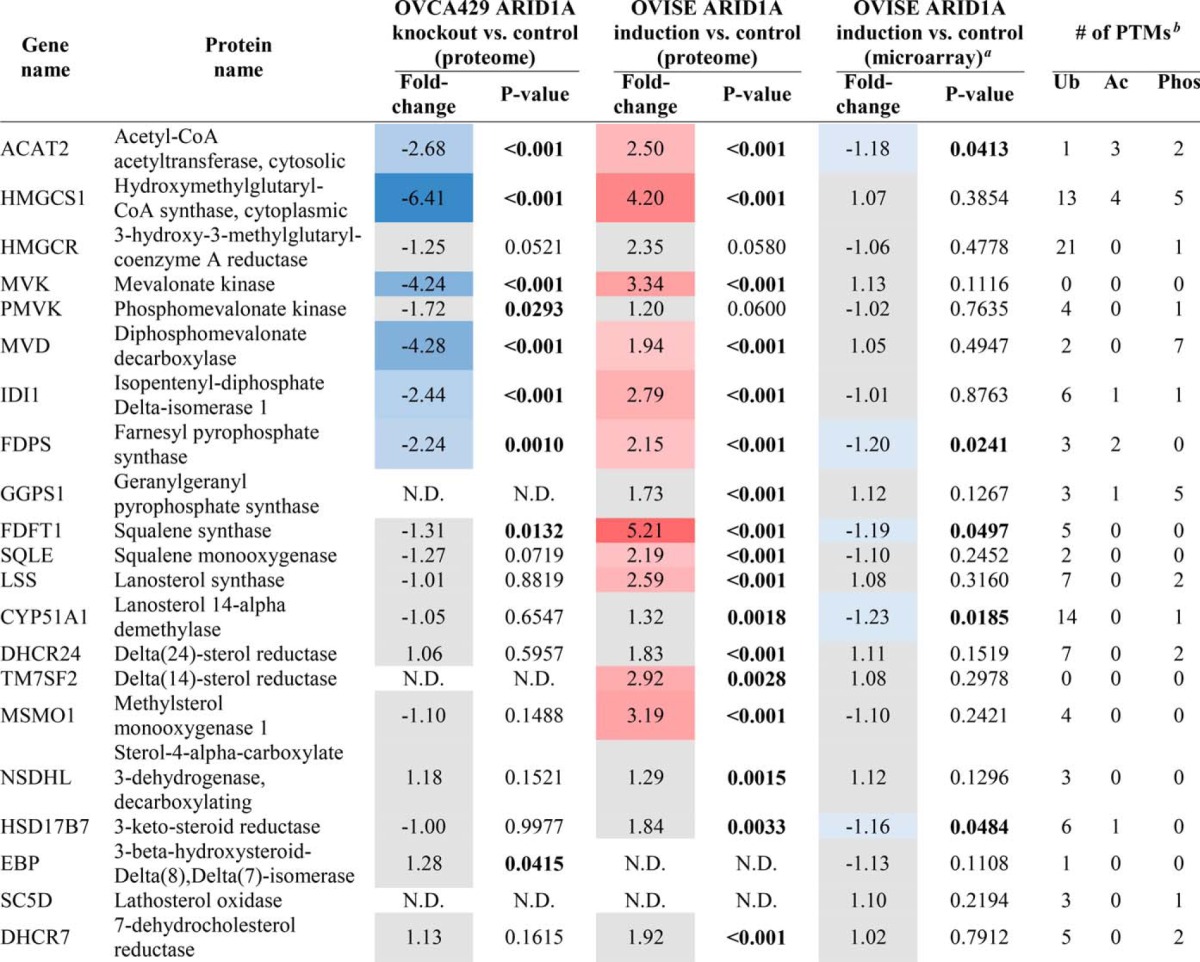
Mevalonate pathway enzymes in proteomics and microarray datasets Changes in abundance of enzymes that participate in mevalonate pathway and related downstream pathways. Inclusion in table based on IPA curation. Refer to supplemental Tables S3 and S6 for protein quantitation. For proteome analyses, significant changes were defined as fold-change > 2 and Student's t-test *p* < 0.05. For enzymes in the proteome analysis that significantly changed in one condition, a slightly relaxed fold-change was allowed in the other condition (i.e. fold-change of 1.8 with Student's *t*-test *p* < 0.05). For the microarray analysis, significant changes were defined as *p* < 0.05. Red and blue background: significantly higher or lower protein/gene level after ARID1A knockout/induction compared to control. Grey background: no significant change in level after ARID1A knockout/induction compared to control. N.D. indicates not detected in a given analysis. Significant *p* values (*p* < 0.05) are bolded.

*^a^* Microarray data were obtained from the Gene Expression Omnibus database, accession number GSE5497 (20).

*^b^* Ubiquitination (Ub), acetylation (Ac), and phosphorylation (Phos) post-translational modifications (PTMs) from Sharpe and Brown, 2013 (52).

##### A Complementary System for Assessing the Proteome Impact of ARID1A Perturbation

We performed a complementary proteomics analysis to validate the effects of ARID1A knockout observed in OVCA429 in an alternative biological context. Wild-type ARID1A was reintroduced into the *ARID1A*-mutated OCCC cell line OVISE (38), which completely lacks expression of ARID1A protein (17), using a tetracycline-inducible construct (supplemental Fig. S3). This experimental approach was recently used to study the transcriptional effects of ectopic expression of wild-type ARID1A in OVISE cells (20).

##### Proteome Analysis of ARID1A Induction in OVISE

We analyzed cell lysates from biological triplicates of ARID1A-induced and control (mutated *ARID1A*) cells by LC-MS/MS on a Thermo Q Exactive HF mass spectrometer using single unfractionated, 4-hour runs. All samples were analyzed together in MaxQuant using a 1% FDR for peptides and proteins, as described above. A total of 66,829 tryptic peptides were identified when acetylation (protein N-terminal) and oxidation (Met) were counted separately from unmodified versions of the 64,245 sequence unique peptides that were identified across all runs (supplemental Table S4). These peptides were assigned to a total of 6,717 protein groups. We further analyzed the set of 5785 protein groups identified by more than a single peptide, which corresponded to an FDR of 0.28% (supplemental Table S5). There was an average of 11 peptides identified per protein group, yielding an average sequence coverage of 30%.

##### Label-free Comparisons Between ARID1A-induced and Control Proteomes

**Fig. 3.**
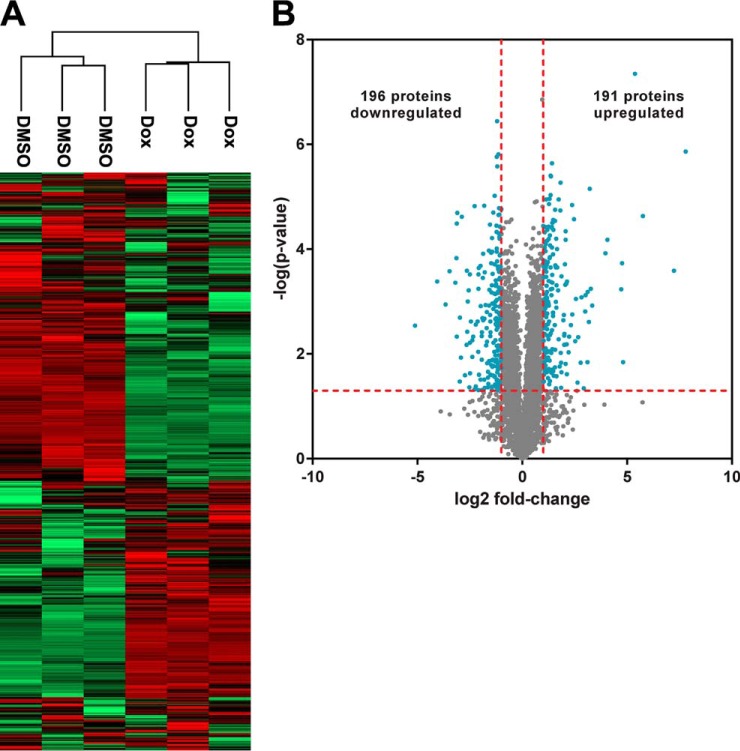
**Characterization of the OVISE proteome with or without induction of wild-type ARID1A expression.** *A*, Two-way, unsupervised hierarchical clustering of OVISE proteomes based on LFQ intensities. *B*, Volcano plot comparing proteomes from ARID1A-induced cells and control cells. Log ratios of LFQ intensities in ARID1A induction *versus* control were plotted against negative log *p* values from the Student's *t* test based on biological triplicates. *Vertical lines*: fold-changes of ± 2. *Horizontal line*: Student's *t* test *p* of 0.05. *Blue points*: proteins that meet both criteria for significant change between ARID1A induction and control (i.e. fold-change in abundance > 2 and *p* < 0.05). *Gray points*: proteins that do not meet both of these criteria.

We performed label-free comparisons between ARID1A-induced and control proteomes. Unsupervised hierarchical clustering resulted in independent clustering based on wild-type ARID1A expression, indicating that ARID1A *s*tatus had a global effect on the proteomes (Fig. 3*A*). The percentage of protein groups that showed ARID1A dependence compared with all identified protein groups was similar in magnitude to the changes observed for the ARID1A knockout. 387 protein groups significantly changed between conditions, corresponding to 7% of the identified proteome (Fig. 3*B*, supplemental Table S6). The cells with induced wild-type ARID1A showed 191 protein groups at higher levels and 196 protein groups at lower levels relative to the control cells. Given that in this system ARID1A is overexpressed relative to a null background, it is unsurprising that the ARID1A protein had the greatest magnitude fold-change among the significantly changed proteins with a 223-fold increase in protein level. In contrast to the ARID1A knockout experiment, the core SWI/SNF complex subunits SMARCB1, SMARCC1, and DPF2 (33) significantly increased in abundance after ARID1A induction. ARID1B, a component of BAF SWI/SNF complexes that is mutually exclusive with ARID1A, and PBRM1, a subunit of the PBAF SWI/SNF complex, both significantly decreased in abundance in response to increased ARID1A levels.

##### ARID1A Induction Up-regulates the Mevalonate Pathway

**Fig. 4.**
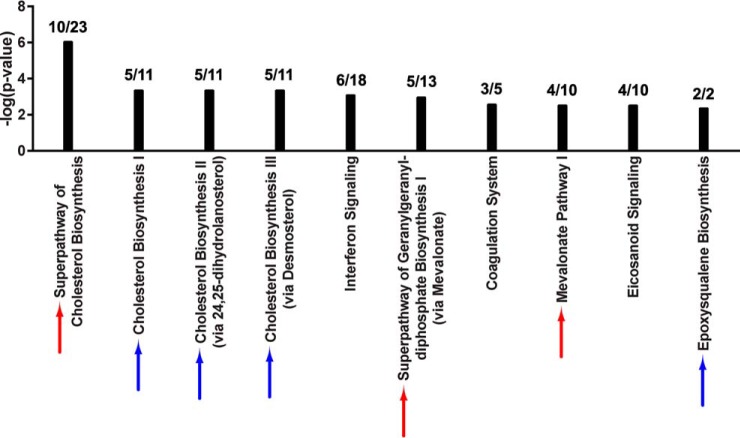
**Induction of ARID1A up-regulates the mevalonate pathway.** Top scoring canonical pathways associated with ARID1A restoration in OVISE are shown (IPA, *p* < 0.005 by Fisher's exact test right-tailed). *Black bars*: negative log *p* values for each canonical pathway. The ratio of annotated proteins that significantly changed in level following ARID1A induction to total identified proteins for a given canonical pathway is shown above each bar. *Red arrows*: canonical pathways that involve the mevalonate pathway and are also significantly enriched among proteins that changed due to ARID1A knockout. Refer to Fig. 2. *Blue arrows*: additional canonical pathways involving the mevalonate pathway.

Pathway analysis was performed on the 378 of the 387 ARID1A-dependent proteins that could be mapped to the IPA database. Seven of the top 10 scoring pathways were associated with the mevalonate pathway: “Superpathway of Geranylgeranyl-diphosphate Biosynthesis I (via Mevalonate),” “Mevalonate Pathway I,” “Superpathway of Cholesterol Biosynthesis,” “Cholesterol Biosynthesis I,” “Cholesterol Biosynthesis II (via 24,25-dihydrolanosterol),” “Cholesterol Biosynthesis III (via Desmosterol),” and “Epoxysqualene Biosynthesis” (Fig. 4). There was extensive coverage of pathway components with 38.5% to 100% of identified pathway proteins significantly changing in abundance, similar to the ARID1A knockout, although the change in protein abundance was in the opposite direction. Specifically, the 10 enzymes that changed among these pathways (ACAT2, HMGCS1, MVK, IDI1, FDPS, FDFT1, SQLE1, LSS1, TM7SF2, and MSMO1) increased in abundance when ARID1A was induced (Table I). All mevalonate pathway-related proteins except HMGCR were identified in all OVISE proteomes or not detected in any sample. HMGCR was detected in a single control proteome (Table S5), therefore two imputed values were used in fold-change and *p* value calculations (supplemental Table S6).

##### Comparison of OVISE Cell Proteomics and Microarray Data

To further explore the basis for the above changes in enzyme levels, we compared the proteomics data to microarray data from OVISE cells ectopically expressing wild-type ARID1A using the same lentivirus transduced cell line (20). Most mevalonate pathway enzymes that significantly increased at the protein level upon ARID1A induction did not show significant changes in gene expression (Table I). For the few genes that significantly changed in level, the magnitude of change was very low. A better concordance between ARID1A-dependent changes in protein and gene levels was observed for proteins that exhibited the greatest changes upon ARID1A induction and their associated protein-coding genes (Table II). Thirteen of the top 25 entries showed consistent protein and gene regulation; however, gene expression changes were much smaller than protein level changes. In some cases, discrepancy in magnitude of change could be because of limitations of using imputed values for proteins that were not detected in either condition. When the effects of imputation were evaluated, 679 proteins groups or 12% of all identified protein groups, were not quantified in at least 1 OVISE proteome (supplemental Table S5) and therefore were assigned imputed values (supplemental Table S6, supplemental Fig. S4). Eight proteins listed in Table II required two or more imputed values for quantitation because of lack of detection in either the ARID1A-induced or control proteomes, though none of these proteins required imputed values for both conditions (Table S7). For example, ARHGDIB had an apparent 10-fold increase in protein abundance compared with a 1.5-fold increase in gene expression following ARID1A induction; however, ARHGDIB protein was not detected in control and, therefore, the calculated fold-change is dependent entirely upon imputed values. Without imputation, fold-change calculations for proteins such as ARHGDIB would result in a division by zero. Approximately 4% of all identified protein groups (204 protein groups) fall into this category and would be classified as being observed in either the ARID1A-induced or control proteomes if imputation had not been used (supplemental Table S5). For proteins present at quantifiable levels in both ARID1A-induced and control proteomes, calculations performed with and without imputation resulted in comparable fold-changes and *p* values (supplemental Table S7). For example, EFNB1 was not detected in two control proteomes. With imputation, the fold-change between ARID1A induction *versus* control was 28.1 with a *p* value of 0.014. With imputation, these values were 20.0 and 0.034, respectively, which still met our criteria for significantly changed proteins.

**Table II:**
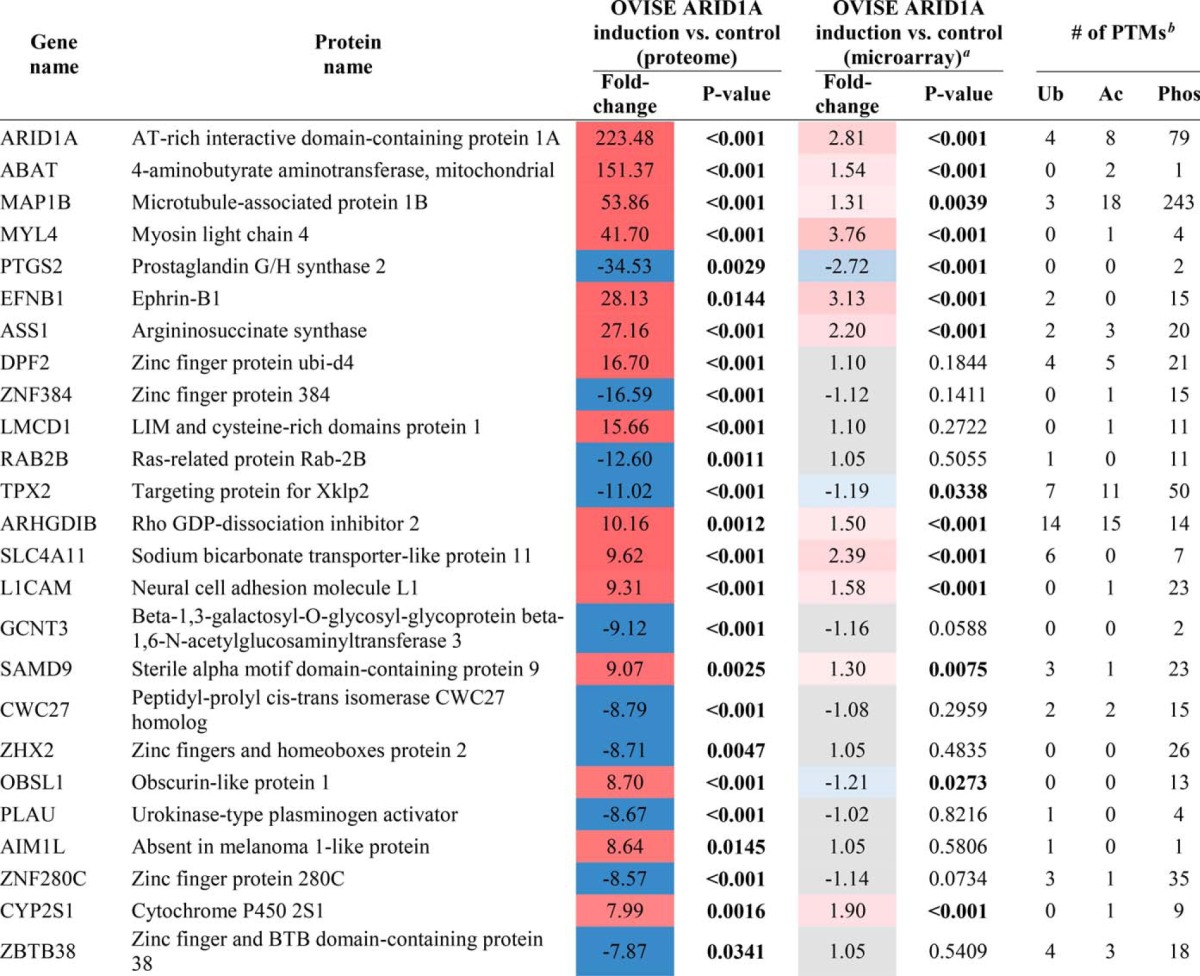
Effects of wild-type ARID1A induction on protein and gene expression levels The 25 proteins showing the largest magnitude, significant changes in protein abundance after ARID1A induction in OVISE cell line. Proteins unable to be mapped to at least one Illumina microarray probe were pre-filtered from the list. Refer to supplemental Table S6 for protein quantitation. For the proteome analysis, significant changes were defined as fold-change > 2 and Student's t-test *p* < 0.05. For the microarray analysis, significant changes were defined as *p* < 0.05. Red and blue background: significantly higher or lower protein/gene level after ARID1A induction. Grey background: no significant change in level after ARID1A induction. N.D. indicates not detected in a given analysis. Significant *p* values (*p* < 0.05) are bolded.

*^a^* Microarray data were obtained from the Gene Expression Omnibus database, accession number GSE5497 (20).

*^b^* Ubiquitination (Ub), acetylation (Ac), and phosphorylation (Phos) post-translational modifications (PTMs) curated by PhosphoSitePlus (http://www.phosphosite.org) (54). Only sites identified in human were considered.

We also searched the proteomics data for known *bona fide* ARID1A targets. SMAD3 and PIK3IP1 (17, 20) were not detected in the proteome data set, while CDKN1A (17) increased moderately, but significantly, in abundance upon ARID1A induction in OVISE (supplemental Table S6).

## DISCUSSION

**Fig. 5.**
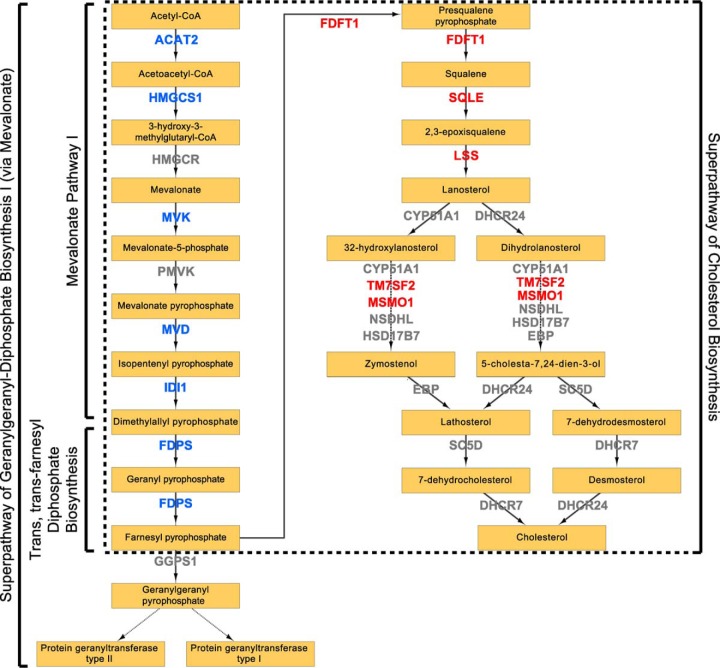
**Regulation of mevalonate pathway by ARID1A.** Schematic diagram of mevalonate pathway based on IPA curation. Canonical pathways overrepresented upon ARID1A knockout in OVCA429 and induction in OVISE are labeled. *Blue*: enzymes significantly decreased in abundance in knockout and significantly increased in abundance in induction. For enzymes that significantly changed in one condition, we allowed for a slightly relaxed fold change in the other condition (*i.e.* 1.8-fold change with Student's *t* test *p* < 0.05). *Red*: enzymes significantly increased in abundance in induction and not significantly changed in knockout. Refer to Table I.

Here, we show that 5–7% of the proteomes in OCCC cell lines significantly change following perturbation of ARID1A expression (Fig. 1*B*, Fig. 3*B*). Despite the relatively moderate global effect on the proteome, the mevalonate pathway and related downstream pathways were associated with both ARID1A knockout in OVCA429 and ectopic expression of wild-type ARID1A in OVISE (Fig. 2, Fig. 4). Examination of specific pathway components revealed that most enzymes in the mevalonate pathway showed abundance changes that correlated with ARID1A status (Table I). That is, most enzymes decreased in abundance in the ARID1A knockout and increased in abundance upon restoration of wild-type ARID1A. Enzymes showing these reciprocal abundance changes include FDPS, which catalyzes production of farnesyl pyrophosphate, a metabolite that is upstream of the branch point that specifies prenylation, cholesterol biosynthesis, and other pathway outputs (Fig. 5). Overexpression of ARID1A is also associated with increased abundance of enzymes in the cholesterol biosynthesis branch downstream of the mevalonate pathway (Table I, Fig. 5). These findings suggest that the mevalonate pathway and related pathways are strongly affected by ARID1A.

Dysregulation of the mevalonate pathway has previously been implicated in cancers and other diseases, mainly due to increased cholesterol biosynthesis and aberrant protein prenylation that occurs when the pathway is spuriously up-regulated (39–41). In breast cancer, mutant p53 up-regulates mevalonate pathway components at least in part through the sterol regulatory element-binding proteins (SREBP) family of transcription factors, and increased mevalonate pathway flux is necessary and sufficient for the mutant p53 phenotype observed in culture (42). Since ARID1A is a tumor suppressor (17), it was surprising that the mevalonate pathway was downregulated in OCCC when ARID1A was knocked out (Table I). Since most oppositely changed enzymes are located in the mevalonate trunk of the pathway before it branches into alternative products (Fig. 5), further study is required to determine the biological importance and relative contribution of the alternative pathway outputs to the OCCC disease phenotype. However, a recently published large Danish demographic study of the linkage between statin use and risk of ovarian cancer showed that OCCC was the only subtype with increased cancer incidence with use of statins and that risk of OCCC increased with length of use (43). This is consistent with downregulation of the mevalonate pathway having an important role in development of OCCC because statins inhibit HMGCR, which catalyzes the rate-limiting step of the pathway (34). This is also consistent with our finding that ARID1A inactivation downregulates the mevalonate pathway.

Given that ARID1A regulates gene transcription through the SWI/SNF complex (17), direct targets of ARID1A should be detectable by monitoring gene expression. However, mevalonate pathway up-regulation after reintroduction of wild-type ARID1A was observed at the protein level but not at the mRNA level (Table I). Although this discrepancy could be because of the fact that separate batches of cells, prepared at different times under somewhat different conditions, were used for the proteome and transcriptome analyses, decoupled responses for groups of genes and proteins are commonly observed when comparing parallel global proteomics and transcriptomics data (44, 45). The better concordance observed between protein and gene expression changes for the 25 proteins with the largest fold-changes (Table II) suggests, but does not prove, that the mevalonate pathway is particularly sensitive to regulation at the post-transcriptional level. A number of studies support this hypothesized post-transcriptional regulation of the mevalonate pathway. Several of these proteins (HMGCR, SQLE, and HMGCS1) have been reported to undergo ubiquitin-regulated proteasomal degradation (46–51). Further, almost all of the other proteins in this pathway have also been reported to be ubiquitinated, and many have been reported to be acetylated or phosphorylated (Table I) (52). Of the proteins that changed in level with ARID1A status, only MVK had no reported modifications. Therefore, the discordance between protein and mRNA changes for the mevalonate pathway when wild-type ARID1A is restored may be because of inhibition of protein degradation through the ubiquitin-proteasome system (53).

Further examination of the 25 proteins with the largest fold-changes illustrates the likely complex and indirect regulation of the proteome by ARID1A (Table II). Similar to mevalonate pathway proteins, DPF2, LMCD1, AIM1L, and OBSL1 showed increased protein levels that did not positively correlate with changes in gene expression. Eleven proteins showed concordant increases in protein and mRNA levels with much larger fold-changes at the protein level (ARID1A, ABAT, MAP1B, MYL4, EFHB1, ASS1, ARHGDIB, SLC4A11, L1CAM, SAMD9, and CYP2S1), consistent with a combinatorial effect of inhibition of protein degradation and activation of gene transcription. However, these mechanisms do not fully explain the relationship between protein and mRNA levels observed among the top 25 proteins. PTGS2 and TPX2 showed concordant decreases in protein and mRNA levels, but the protein changes were an order-of-magnitude greater. Further, eight proteins (ZNF384, RAB2B, GCNT3, CWC27, ZHX2, PLAU, ZNF280C, and ZBTB38) did not display significant changes in mRNA levels but had large decreases at the protein level. Six of these proteins have reported ubiquitination sites, and the other two proteins are known to be either acetylated or phosphorylated (Table II), suggesting that the basis for the decreased protein levels could be due to increased protein degradation through ubiquitination or changes in other post-translational modifications. These observations suggest that ARID1A may inhibit degradation of some proteins and promote degradation of other proteins possibly by differentially affecting specific ubiquitin ligases. Further studies are required to identify specific targets of ARID1A involved in post-transcriptional regulation, including potential changes in protein degradation as postulated here.

This study is, to the best of our knowledge, the first in-depth proteome analysis addressing the role of ARID1A in OCCC. Protein level changes rather than transcriptional changes best reflect the state of the cell when wild-type ARID1A is knocked out or restored. Identification of the mevalonate pathway as the primary target of ARID1A loss of function suggests areas for further investigation and potential therapeutic targeting for *ARID1A*-mutated cancers.

## Supplementary Material

Supplemental Data

## Footnotes

^1^ The abbreviations used are:

OCCC: Ovarian clear cell carcinoma
BAF: BRG1-associated factor
CRISPR: Clustered regularly interspaced short palindromic repeats
FDR: False discovery rate
LFQ: Label-free quantitation
PBAF: Polybromo BRG1-associated factor
PTM: Post-translational modification
SREBP: Sterol regulatory element–binding protein
SWI/SNF: Switch/sucrose non-fermentable.
