# Supplementary material for: The Primary Effect on the Proteome of ARID1A-mutated Ovarian Clear Cell Carcinoma is Downregulation of the Mevalonate Pathway at the Post-transcriptional Level
Source: Mol Cell Proteomics. 2016 Sep 21;15(11):3348–60. doi: 10.1074/mcp.M116.062539 (PMC5098034; doi:10.1074/mcp.M116.062539)
Supplement: Supplemental Data [file supp_15_11_3348__index.html]

The Primary Effect on the Proteome of ARID1A-mutated Ovarian Clear Cell Carcinoma is Downregulation of the Mevalonate Pathway at the Post-transcriptional Level — Effect of ARID1A Loss in Ovarian Clear Cell Carcinoma — Supplemental Data 

# The Primary Effect on the Proteome of *ARID1A*-mutated Ovarian Clear Cell Carcinoma is Downregulation of the Mevalonate Pathway at the Post-transcriptional Level

## Supplemental Data

- Supplemental Table S1 (.xlsx, 9.2 MB) - Supplemental Table S1. Peptide identifications in biological triplicate analyses of OVCA429 with and without ARID1A CRISPR knockout.
- Supplemental Table S2 (.xlsx, 1.2 MB) - Supplemental Table S2. Protein identifications in biological triplicate analyses of OVCA429 with and without ARID1A CRISPR knockout.
- Supplemental Table S3 (.xlsx, 1.1 MB) - Supplemental Table S3. Quantitative proteome comparisons between OVCA429 with and without ARID1A CRISPR knockout.
- Supplemental Table S4 (.xlsx, 11.3 MB) - Supplemental Table S4. Peptide identifications in biological triplicate analyses of OVISE with and without ARID1A induction.
- Supplemental Table S5 (.xlsx, 1.4 MB) - Supplemental Table S5. Protein identifications in biological triplicate analyses of OVISE with and without ARID1A induction.
- Supplemental Table S6 (.xlsx, 1.3 MB) - Supplemental Table S6. Quantitative proteome comparisons between OVISE with and without ARID1A induction.
- Supplemental Data (.pdf, 774 KB) - Supplemental Figures S1-S4 and Supplemental Table S7.
