# Supplementary material for: The Primary Effect on the Proteome of ARID1A-mutated Ovarian Clear Cell Carcinoma is Downregulation of the Mevalonate Pathway at the Post-transcriptional Level
Source: Mol Cell Proteomics. 2016 Sep 21;15(11):3348–60. doi: 10.1074/mcp.M116.062539 (PMC5098034; doi:10.1074/mcp.M116.062539)
Supplement: Supplemental Data [file 10.1074_M116.062539_mcp.M116.062539-7.pdf]

Supplemental Data

Figure S1. Validation of ARID1A knockout in OVCA429. Representative immunoblot of cell lysates from ARID1A CRISPR knockout or control cells derived from OVCA429. Two CRISPR knockout clones are shown. Clone #2 had a somewhat stronger response to the EZH2 inhibitor GSK12 (20) and was used for the detailed proteome analysis summarized in this study.

Figure S2. Reproducibility of OVCA429 proteome analysis. A-B. Scatterplots of LFQ intensities for proteins identified in OVCA429 proteomes from two passages of control (A) and knockout (B) cells. R^2 calculated from linear-fit trend line.

Figure S3. Validation of ARID1A induction in OVISE. Representative immunoblot of cell lysates from OVISE cell line harboring a tetracycline-inducible wild-type ARID1A construct treated with vehicle (DMSO) or doxycycline to induce expression (DOX).

Figure S4. Distribution of LFQ intensities for protein groups identified in proteome analyses. Histograms of protein group LFQ intensities are shown for OVCA429 proteomes (A) and OVISE proteomes (B). Blue bars: frequency of LFQ intensities calculated by MaxQuant. Orange bars: frequency of LFQ intensities imputed by Perseus.

Supplemental Table S1. Peptide identifications in biological triplicate analyses of OVCA429 with and without ARID1A CRISPR knockout.

Supplemental Table S2. Protein identifications in biological triplicate analyses of OVCA429 with and without ARID1A CRISPR knockout.

Supplemental Table S3. Quantitative proteome comparisons between OVCA429 with and without ARID1A CRISPR knockout.

Supplemental Table S4. Peptide identifications in biological triplicate analyses of OVISE with and without ARID1A induction.

Supplemental Table S5. Protein identifications in biological triplicate analyses of OVISE with and without ARID1A induction.

Supplemental Table S6. Quantitative proteome comparisons between OVISE with and without ARID1A induction.

Supplemental Table S7. Effects of wild-type ARID1A induction on protein levels with and without imputation of missing values. Calculation of fold-change and p-values for proteins listed in Table 2 with or without replacing missing values by imputation. With imputation, log ratios and Student's t-test p-values were calculated using $\log_2$ transformed data as described in Experimental Procedures and converted into fold-changes. Without imputation, missing values were kept as 0, and fold-changes and Student's t-test p-values were calculated using untransformed data. If a protein was only quantified in one condition, a division by zero error occurred ("DIV/0"). Red and blue background: higher or lower protein level after ARID1A

induction. Grey background: no significant change in level after ARID1A induction. Significant p-values ($p < 0.05$) are bolded.

Gene name	Protein name	OVISE ARID1A induction vs. control (with imputation)		# missing values		OVISE ARID1A induction vs. control (without imputation)	
		Fold-change	P-value	ARID1A Induction	Control	Fold-change	P-value
ARID1A	AT-rich interactive domain-containing protein 1A	223.48	**<0.001**	0	1	349.17	**<0.001**
ABAT	4-aminobutyrate aminotransferase, mitochondrial	151.37	**<0.001**	0	0	131.27	**<0.001**
MAP1B	Microtubule-associated protein 1B	53.86	**<0.001**	0	0	52.26	**<0.001**
MYL4	Myosin light chain 4	41.70	**<0.001**	0	0	41.72	**<0.001**
PTGS2	Prostaglandin G/H synthase 2	-34.53	**0.0029**	1	0	-29.20	**<0.001**
EFNB1	Ephrin-B1	28.13	**0.0144**	0	2	20.03	**0.0342**
ASS1	Argininosuccinate synthase	27.16	**<0.001**	0	0	25.84	**<0.001**
DPF2	Zinc finger protein ubi-d4	16.70	**<0.001**	0	0	16.39	**<0.001**
ZNF384	Zinc finger protein 384	-16.59	**<0.001**	3	0	DIV/0	**<0.001**
LMCD1	LIM and cysteine-rich domains protein 1	15.66	**<0.001**	0	0	15.64	**0.0019**
RAB2B	Ras-related protein Rab-2B	-12.60	**0.0011**	0	0	-13.85	**0.0475**
TPX2	Targeting protein for Xklp2	-11.02	**<0.001**	0	0	-11.42	**0.0110**
ARHGDIB	Rho GDP-dissociation inhibitor 2	10.16	**0.0012**	0	3	DIV/0	**0.0060**
SLC4A11	Sodium bicarbonate transporter-like protein 11	9.62	**<0.001**	0	2	22.79	**0.0052**
L1CAM	Neural cell adhesion molecule L1	9.31	**<0.001**	0	0	9.30	**<0.001**
GCNT3	Beta-1,3-galactosyl-O-glycosyl-glycoprotein beta-1,6-N-acetylglucosaminyltransferase 3	-9.12	**<0.001**	0	0	-8.84	**<0.001**
SAMD9	Sterile alpha motif domain-containing protein 9	9.07	**0.0025**	0	0	8.73	**0.0068**
CWC27	Peptidyl-prolyl cis-trans isomerase CWC27 homolog	-8.79	**<0.001**	3	0	DIV/0	**<0.001**
ZHX2	Zinc fingers and homeoboxes protein 2	-8.71	**0.0047**	3	0	DIV/0	**0.0170**
OBSL1	Obscurin-like protein 1	8.70	**<0.001**	0	0	8.35	**<0.001**
PLAU	Urokinase-type plasminogen activator	-8.67	**<0.001**	0	0	-8.63	**<0.001**
AIM1L	Absent in melanoma 1-like protein	8.64	**0.0145**	0	0	7.97	**0.0349**
ZNF280C	Zinc finger protein 280C	-8.57	**<0.001**	3	0	DIV/0	**<0.001**
CYP2S1	Cytochrome P450 2S1	7.99	**0.0016**	0	0	7.45	**<0.001**
ZBTB38	Zinc finger and BTB domain-containing protein 38	-7.87	**0.0341**	2	0	-7.17	**0.0253**

Fig. S1

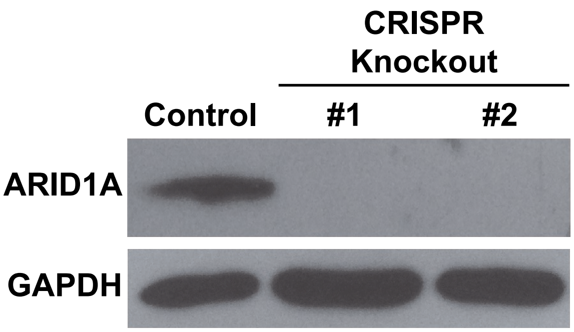

Fig. S2

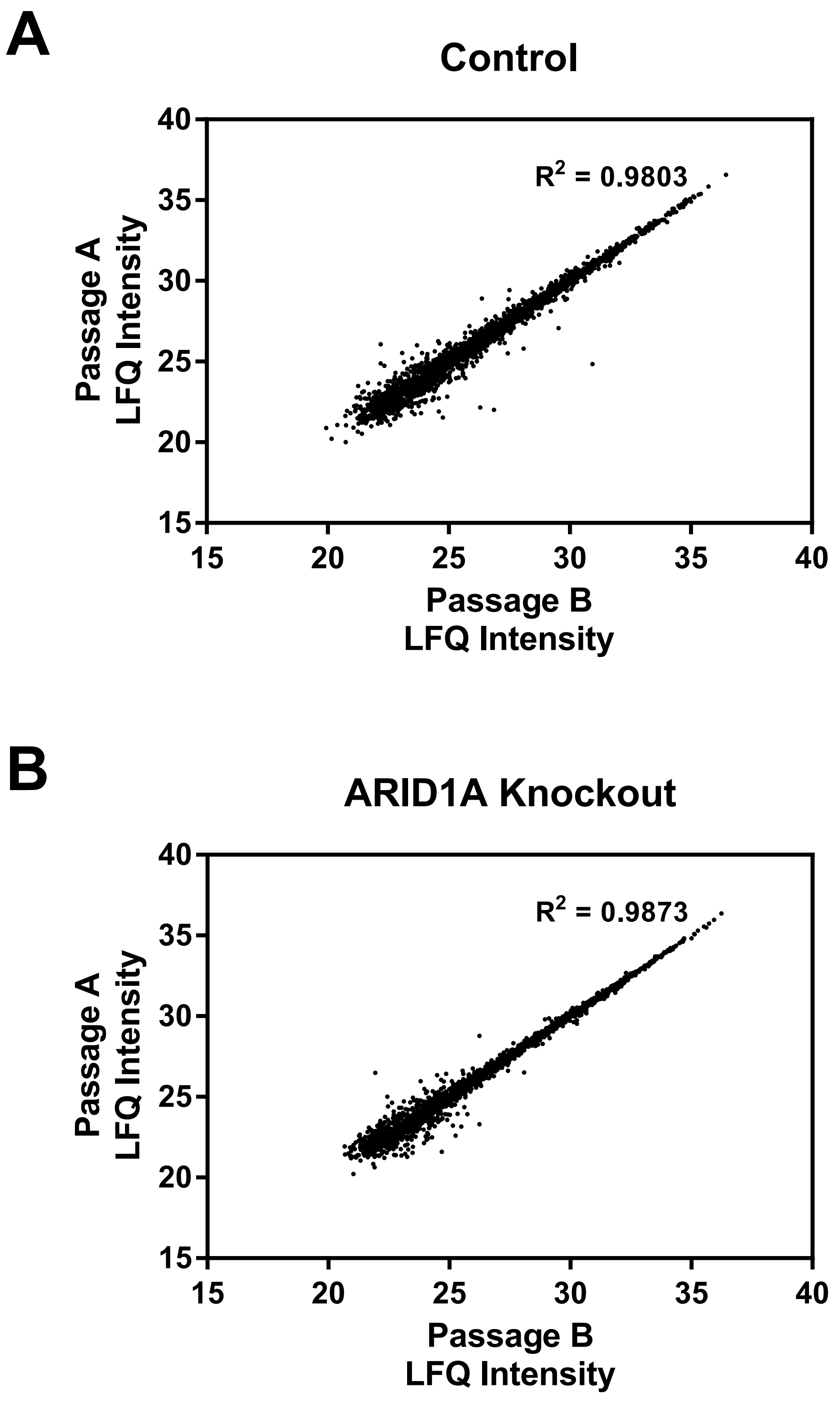

A
Control
R^2 = 0.9803
Passage A LFQ Intensity
Passage B LFQ Intensity
15
20
25
30
35
40
B
ARID1A Knockout
R^2 = 0.9873
Passage A LFQ Intensity
Passage B LFQ Intensity

Fig. S3

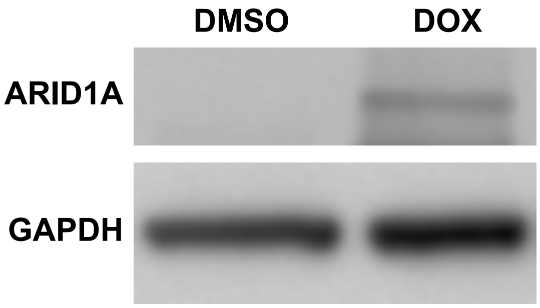

Fig. S4

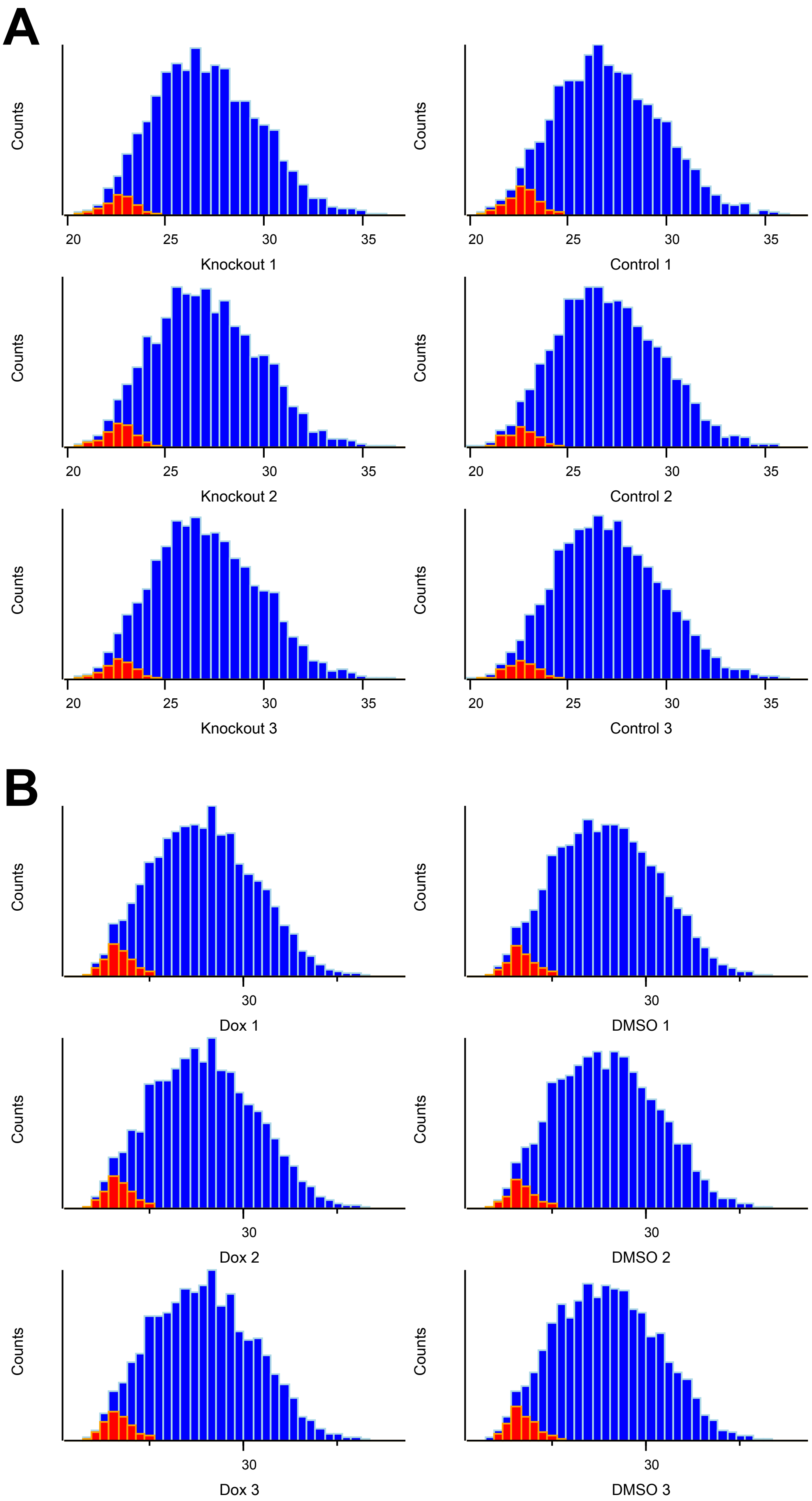
A
Counts
20
25
30
35
Knockout 1
Control 1
Knockout 2
Control 2
Knockout 3
Control 3
B
30
Dox 1
DMSO 1
Dox 2
DMSO 2
Dox 3
DMSO 3
